# Group and individual variability in speech production networks during delayed auditory feedback

**DOI:** 10.1121/1.5026500

**Published:** 2018-05-22

**Authors:** Z. K. Agnew, C. McGettigan, B. Banks, S. K. Scott

**Affiliations:** Institute for Cognitive Neuroscience, University College London, 17 Queen Square, London WC1N 3AR, United Kingdom

## Abstract

Altering reafferent sensory information can have a profound effect on motor output. Introducing a short delay [delayed auditory feedback (DAF)] during speech production results in modulations of voice and loudness, and produces a range of speech dysfluencies. The ability of speakers to resist the effects of delayed feedback is variable yet it is unclear what neural processes underlie differences in susceptibility to DAF. Here, susceptibility to DAF is investigated by looking at the neural basis of within and between subject changes in speech fluency under 50 and 200 ms delay conditions. Using functional magnetic resonance imaging, networks involved in producing speech under two levels of DAF were identified, lying largely within networks active during normal speech production. Independent of condition, fluency ratings were associated with midbrain activity corresponding to periaqueductal grey matter. Across subject variability in ability to produce normal sounding speech under a 200 ms delay was associated with activity in ventral sensorimotor cortices, whereas ability to produce normal sounding speech under a 50 ms delay was associated with left inferior frontal gyrus activity. These data indicate whilst overlapping cortical mechanisms are engaged for speaking under different delay conditions, susceptibility to different temporal delays in speech feedback may involve different processes.

## I. INTRODUCTION

### A. Speech production is highly sensitive to the auditory information

Speech production is highly sensitive to context: speakers modulate their vocal behaviour according to both the auditory environment, and also to their own reafferent feedback. In noisy environments, speakers unconsciously alter various aspects of the voice, including raising the volume (Lane and Tranel, 1971; Junqua, 1993), flattening of spectral tilt (more energy at higher frequencies), as well as changes to F0 and F1 (Lu and Cooke, 2009; Cooke and Lu, 2010). In addition to background noise and context, reafferent feedback also plays an important role in certain aspects of speech motor control. Post-lingually deaf individuals display alterations to both segmental and suprasegmental aspects of speech, such as dysfluencies and reduced speech rate (Cowie *et al.*, 1982; Lane and Webster, 1991; Schenk *et al.*, 2003). Studies that manipulate auditory or somatosensory feedback during speech indicate that speakers also modify their speech according to reafferent information (MacKay, 1970; Fabbro and Daro, 1995; Hashimoto and Sakai, 2003; Jones and Striemer, 2007), suggesting at least some degree of influence of sensory feedback during speech. By quantifying behavioural and neural responses to manipulations of sensory feedback during speech motor control, these studies have revealed some of the mechanisms involved in the sensory control of speech (Yates, 1963; Larson *et al.*, 2000; Nasir and Ostry, 2009; Patel *et al.*, 2011; Kort *et al.*, 2014). Susceptibility to alterations in feedback reveals aspects of the role of sensory processing during motor control of speech. For example, rapid compensatory responses to altered F0 provide a biological marker for feedback sensitivity in vocal control of pitch (Jones and Munhall, 2000; Kort *et al.*, 2014). Similarly, delayed auditory feedback (DAF) (the induction of a temporal asynchrony between speech motor commands and auditory feedback) reveals a sensitivity to temporal aspects of auditory feedback. In relation to vocal behaviour, DAF results in dysfluent speech which manifests itself in a range of speech errors that fall into four major categories; time, rate, fluency, and articulation (Fairbanks, 1955; Webster and Dorman, 1971). Overreliance on reafference information is thought to play a role in stuttering, which shares many behavioral similarities to DAF affected speech (Grafton *et al.*, 1997). Interestingly, introducing a delay in feedback, which perhaps modifies any overreliance on reafferent information, is also known to improve speech fluency in people who stutter (Foundas *et al.*, 2004).

### B. The role of feedback in vocal motor control

In humans, alterations to speech feedback in pitch, spectrum or timing affect a talker's speech. Such feedback disruptions, lead to speech dysfluency in many individuals (Lee, 1950; Black, 1951; Langova *et al.*, 1970; Siegel *et al.*, 1982; Fukawa *et al.*, 1988; Stager and Ludlow, 1993).The profound effects that perturbation of sensory information such as DAF has on speech production is often interpreted as evidence that auditory feedback is used to monitor speech production in a closed loop manner (Lee, 1950; Fairbanks, 1955). Previous studies have demonstrated that a 50 ms delay in feedback is detectable by the speaker, but does not result in maximal interference of speech production (Black, 1951); maximal interruption is seen around a 200 ms delay for most speakers (Takaso *et al.*, 2010), irrespective of the length of speech sounds (Farrow *et al.*, 2001). Subjects report finding speech harder to produce as the delay length increases, and perceived accuracy of articulation is reduced as delay length increases (Takaso *et al.*, 2010). Together this suggests that 200 ms is a critical DAF interval, independent of speech rate or the length of speech reafferent sounds. Neuroimaging has revealed that producing speech under altered auditory feedback conditions compared to normal feedback is associated with activity in bilateral temporal parietal regions (Hirano *et al.*, 1997; Hashimoto and Sakai, 2003; Fu *et al.*, 2006). Increased activity in the superior temporal cortices during speech under altered auditory feedback has been shown to be independent of speech rate, correlated with the severity of 200 ms DAF effects on speech (Hashimoto and Sakai, 2003), and negatively associated with misattributions of one's voice to an external source (Fu *et al.*, 2006).

At a cortical level, there is considerable evidence that the response to a speakers own voice during speech production is reduced in dorsolateral temporal regions, a phenomenon known as sensory suppression (Wise *et al.*, 1999; Houde *et al.*, 2002; Agnew *et al.*, 2013). This has been well documented both in human speech (Houde *et al.*, 2002; Agnew *et al.*, 2013) as well as in non-human primates during vocal behavior (Eliades and Wang, 2003, 2005). This neural phenomenon is known as speech or vocalization induced suppression, one manifestation of a more general motor induced suppression that is seen in response to self generated sensory input (Blakemore *et al.*, 1998, 2000).

### C. Alterations to auditory feedback modulates sensory induced suppression

In other studies looking at altered auditory feedback, suppressed responses in auditory cortex are released from suppression. Thus, we see an increased response in auditory cortex during altered compared to unaltered feedback trials, a neural phenomenon known as speech perturbation response enhancement (SPRE) which has been observed in humans (Chang *et al.*, 2013; Kort *et al.*, 2014) and nonhuman primates (Eliades and Wang, 2008). This enhanced response has been localized to specific ventral premotor and temporal sites using electrocorticography and other neuroimaging techniques (Kort *et al.*, 2014). Vocal compensatory behavior is predicted by the magnitude of neural responses to altered auditory feedback, more so in sites displaying SPRE responses (Chang *et al.*, 2013). Together these studies imply a highly tuned relationship between vocal behavior and these neural phenomena (vocalization induced suppression, and SPRE), suggesting that they may place a central role in corrective vocal motor control. It is clear then, that the investigation of the relationship between individual susceptibility to altered auditory feedback and the corresponding neural responses will inform theories of speech motor control greatly.

### D. The effects of altering feedback is variable across subjects

It is well established that there is a wide range in individual susceptibility to the DAF effect: performance under DAF is associated with a wide range of both within subject (Burke, 1975) and across subject variability (Yates, 1963). For high susceptibility individuals, speech is rendered unintelligible where as others remain relatively impervious to the effects of DAF. DAF disrupts the speech of children more than that of adults, regardless of length of delay (Smith and Tierney, 1971; Farrow *et al.*, 2001; Gallese *et al.*, 2002). Adults speaking in their less fluent language have also been shown to display greater DAF interference effects (MacKay, 1987). Beyond this, personality traits such as self-percept stability and paranoid tendencies have been found to be correlated with increased variation in vocal intensity in response to DAF (Spilka, 1954). It has been suggested, for example, that speakers showing extreme susceptibility to DAF may be differentially dependent on auditory feedback in regulating their speech production (Yates, 1963, 1965) whereas others have shown that susceptibility is linked to coping strategies (Burke, 1975). Other attempts to look at correlation in performance under DAF and language abilities (Arens and Popplestone, 1959) or normal speech performance (Butler and Galloway, 1957) have not found conclusive evidence for specific factors. Neurally, it has recently been shown that intersubject variability in brain activity reflects meaningful changes rather than noise (Amunts and Willmes, 2006). Together these data suggest that a rage of factors may contribute to a greater susceptibility to DAF and that an understanding the role of sensory and motor networks in governing individual sensitivity to DAF, is an essential part of understanding the role of temporal feedback control in speech production.

Here we aim to specifically investigate the neural underpinnings of this variability in ability to produce normal speech, both within and across subjects, under two delay conditions (200 and 50 ms delay), and under normal feedback. We aim to explore and distinguish between the neural response to producing speech with DAF, the neural activity that correlates with the length of this delay and the neural activity that correlates with susceptibility to these different delay lengths. Specifically, we aim to investigate the following.

(1) Whether the neural correlates of producing speech under these two different delays is overlapping, and distinct only in magnitude of response.
(2) Whether behavioral measures of ability to speak normally under both conditions is correlated within subjects.
(3) Whether the pattern of BOLD responses associated with ability to speak normally under both conditions is overlapping or distinct.

In order to address these questions, we used functional magnetic resonance imaging to look at BOLD responses during speech production under DAF (with a delay of 200 and 50 ms), and under normal feedback conditions (0 ms delay). Produced speech was assessed for fluency and these measures were used to investigate the neural networks underlying individual susceptibility to interference from DAF.

## II. METHODS

### A. Stimuli

In order to construct all the required conditions, we required auditory recordings from a corpus and visually presented sentences from the same corpus for motor output conditions. All stimuli were generated from the Institute of Electrical and Electronic Engineers (IEEE) corpus (1969), for example, “The birch canoe slid on the smooth planks.” In order to make the auditory stimuli for the silent articulation with listening condition, sentences were produced by a variety of speakers. All speech stimuli were produced by native British speakers which comprised both male and female speakers with a range of regional accents. We used speech recorded from a range of British speakers such that everybody heard the same male and female speakers. Text was presented using psychophysics toolbox running on matlab 7.4 (Mathworks, Inc., Sherborn, MA). Speech stimuli were recorded using a solid state recorder (Edirol, R-09HR) at 24 bits, 96 kHz, and saved as wav files. The sound files were normalized using the peak amplitude in praat (Boersma and Weenink, 2010). Sentences comprised 30 sentences which were repeated for each condition.

### B. Subjects

Twenty healthy right-handed subjects (mean age 26 years +/− 5, 11 female) participated in the present study. All were native English speakers and we excluded any subjects who had any history of speech or hearing deficits. All gave informed consent according to the guidelines approved by UCL Ethics Committee who provided local ethics approval for this study.

### C. Conditions

The present experiment involved five conditions: speaking under normal feedback (DAF0), under a 50 ms delay (DAF50), a 200 ms delay (DAF200), passive listening to the same sentences (Listen), and rest (Read). In the rest condition, text was presented on the screen but subjects were instructed to remain silent. Each sentence was presented multiple times, once for each condition. Text was presented in a pseudorandomized order using Psychophysics toolbox running on matlab with the psychophysics toolbox extension (Brainard, 1997).

### D. fMRI

A 1.5 Tesla Siemens Avanto system (Siemens AG, Erlangen, Germany) in combination with a 12-channel head coil was used to acquire 180 T2*-weighted whole brain echo-planar images (EPI) data (3 × 3 × 3 mm^3^ in-plane resolution, TR/TA/TE/flip 9000 ms/3 s/50 ms/90°, 35 slices). A sparse-sampling routine (Hall *et al.*, 1999) was employed, in which sentences were read aloud from visually presented sentences in the quiet period between scans.

**FIG. 1.**
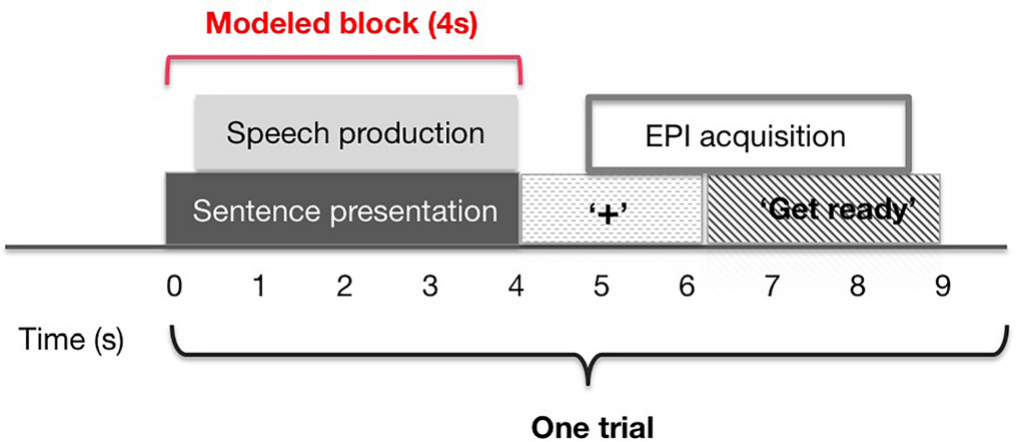
(Color online) Experimental setup. Each 9 s trial consisted of a 3 s instruction, visually presented on a black screen “Get ready to speak/rest/listen.” Sentence presentation began at the onset of the silent period between EPI acquisition, and speech production began soon after sentence presentation. After 4 s, the sentence was replaced with a fixation cross indicating the subject to relax. At 5 s after sentence presentation, a single EPI volume was acquired.

Each event comprised a visual presented instruction followed by the presentation of one sentence during a 4 s period of silence during which time they would read the sentence aloud (Fig. 1). The instruction did not indicate whether the subsequent trial would involve a delay in feedback or not. After the 4 s gap the text on the screen was replaced with a fixation cross to indicate the end of each trial, which coincided with onset of the whole-brain volume. Following a listen instruction, the same text would appear on the screen and the subject would hear the sentence on the screen being read aloud. Following a “Rest” instruction, the same text would appear on the screen and subjects were to silently read the sentence but remain silent. Whilst subjects were informed that the experiment was looking at speech production and practised reading aloud in the pre-scan training, all subjects were naive to the inclusion of DAF conditions until they experienced them in the scanner. There were 30 examples of each of the six conditions presented in a pseudorandomized order. The functional run lasted approximately 27 min (6 conditions × 30 trials × 9 s TR).

### E. Stimulus presentation

Stimuli were presented using matlab with the psychophysics toolbox extension. The audio channel was routed through a Sony HD-510 amplifier (Sony Europe Limited, Weybridge, UK) to electrodynamic MR-compatible headphones worn by the participant (Sensimetrics Corporation, Malden, MA). Instructions were presented via front-projection from an EIKI LC-XG250 projector (Eiki International, Inc., Rancho Santa Margarita, CA) to a custom-built screen at the mouth of the scanner bore, which was viewed using a mirror placed on the head coil. Instructions were projected from a specially configured video projector (Eiki International, Inc., Rancho Santa Margarita, CA) onto a custom-built front screen, which the participant viewed via a mirror placed on the head coil. Speech output was recorded using audacity (2015).

### F. Altered auditory feedback

DAF were presented using matlab with the psychophysics toolbox extension (Brainard, 1997), via a Denon amplifier (Denon UK, Belfast, UK) and electrodynamic headphones worn by the participant (MR Confon GmbH, Magdeburg, Germany).

### G. Pre-processing and analyses

Functional data were analyzed using SPM8 (Wellcome Department of Imaging Neuroscience, London, UK) running on matlab 7.4 (Mathworks, Inc., Sherborn, MA). Functional images were realigned and unwarped, coregistered with the anatomical image, normalized using parameters obtained from unified segmentation of the anatomical image, and smoothed using a Gaussian kernel (8 mm full width at half maximum).

#### 1. First and second level models

At the single-subject level, events lasting 4 s starting from the presentation of the text prompt, were modelled for all four conditions, using a canonical hemodynamic response function in SPM8, along with six movement parameters of no interest. Contrast images for each condition against the rest condition were calculated in the single subject and taken forward to a second-level, random effects flexible factorial analysis of variance (ANOVA) model in SPM8, with factors Subject × Condition. From this model, F contrast images were calculated for the Main Effect of Delay (0, 50, 200 ms), as well as T-contrasts describing each condition compared to each other. All second-level models were calculated at a voxelwise threshold of p < 0.005 (uncorrected), with a voxel threshold of 20 voxels to limit potential type II errors.

#### 2. Conjunction analyses

A conjunction null (Nichols *et al.*, 2005) identifies voxels that are significantly active in more than one contrast. This is done by taking the intersection mask of two thresholded images so that it is possible to look at voxels that are significantly active in the contrast (A > B) and also in the contrast (C > D). These were carried out using a masking threshold of p < 0.001. Significant BOLD effects were rendered on a normalized template. In the present study a conjunction null was calculated between DAF200 > 50 and DAF50 > DAF0. This approach identified voxels significantly more active during speech produced under a 200 ms delay compared to 50 ms delay, and also significantly more active for 50 ms delay compared to no delay. This identified voxels active during increasing delay compared to a shorter delay, at two different delay conditions, thus revealing active regions that are sensitive to increasing delay.

#### 3. Region of interest analyses

Region of interest analyses were carried out to investigate mean effect sizes in specific regions across all experimental conditions against baseline, using the marsbar toolbox that is available for use within SPM8 (Brett *et al.*, 2002). Regions of interest were selected *post hoc*, using peaks from contrasts of interest to investigate the profile of activity in these regions across other conditions. Statistical comparisons were not applied to the extracted effect sizes so as to avoid “double dipping” (Kriegeskorte *et al.*, 2009). Second-level clusters were used to extract condition-specific parameter estimates from regions of interest (using marsbar, Brett *et al.*, 2002). The anatomical locations of peak and sub-peak voxels (at least 8 mm apart) were labelled using the spm anatomy toolbox (version 20) (Eickhoff *et al.*, 2005).

### H. Behavioural testing

Speech produced in the scanner was recorded in order that it be assessed for normalcy: the ability of each individual to produce normal sounded speech under DAF conditions. For each subject, audio recordings of each trial were assessed by three phonetically trained raters. During this assessment, for each sentence, the text that the subject had been presented with, was presented on a screen to the assessors, just as it had for the subject during the scan. One second later the audio recording was played through headphones (Technics, Panasonic). Raters were then asked to make a rating via a button press. All raters were blinded to the conditions for each of the stimuli, and to the participants. They were asked to assess the sentences, with the instruction, “How normal do you think this speech sounds? For normal speech give a score of 9 and for completely abnormal or incorrect speech, please score a 1.” Raters were instructed to categorise slowing, slurring, stopping and starting, changes to timing or incorrect words as abnormal, in addition to unusual patterns of pitch and loudness. Behavioural measures of normalcy were obtained for 15 subjects (audio recordings for five subjects were lost during acquisition). A mean normalcy score (1 to 9) for each subject was calculated for across trial variability analyses (see below). For investigating within-subject individual analyses, behavioural scores for each trial were used to make linear parametric modulators across all trials, across all DAF conditions, for each subject. In order to look at variability in performance, two different analyses were performed.

(1) Within subjects: In order to look at the neural differences underlying production of normal sounding across all three speaking conditions, linear parametric modulators were entered in at the first level (see earlier for details on how parametric modulators were created). This approach revealed the neural control of producing normal sounding sentences across all DAF conditions.
(2) Between subjects: In order to look at the neural differences underlying the ability to speak fluently under the three different experimental conditions, mean normalcy scores were entered into a second level model.

For both of these analyses a threshold of p < 0.005 was employed with a cluster threshold of 20. Significant BOLD effects were rendered on a normalized template. Region of interest analyses were carried out to investigate mean effect sizes in specific regions across all experimental conditions against baseline, using the marsbar toolbox that is available for use within SPM8 (Brett *et al.*, 2002).

## III. RESULTS

### A. Behavioral scores of speech output

A one way repeated measures ANOVA revealed significant differences between the scores assigned to sentences spoken under the three different conditions of DAF200, DAF50, and no delay [F(2,42) = 42.852, p < 0.001] means/standard deviations 3.59 +/− 1.94, 5.14 +/− 2.0, 8.20 +/− 1.18, respectively). A Games-Howell *post hoc* test revealed that the scores for all three conditions are significant different from each other.

A product-moment correlation coefficient was computed to assess the relationship between mean scores across all three conditions. This approach revealed significant correlations between the subject mean scores on the three conditions. Speech produced under normal feedback and under DAF50 conditions was highly correlated [r = 0.91, n = 15, p < 0.05) indicating a strong positive relationship between ability to produce normal speech under DAF50 and normal sounding speech under normal feedback conditions. Subject mean ratings of speech produced under DAF200 were correlated with DAF50 speech ratings [r = 0.52, n = 15, p < 0.05) indicating a moderate positive relationship between ability to produce normal speech under the two delay conditions. Speech produced under normal feedback and under DAF200 conditions was the least correlated [r = 0.32, n = 15, p < 0.05) indicating only a weak to moderate relationship between normal sounding speech under no delay and the delay with maximal interference. This indicates that subjects who produced fluent, clear speech under conditions of no delay, also tended to produce more normal sentences under both the DAF conditions (see Table I).

**TABLE I.** Bivariate Pearson's correlation demonstrates scores are correlated across the three conditions. In order to look at the relationship between performance on each of the three speech production conditions, a bivariate Person's correlation test was carried out on the means scores for all 15 subjects. This revealed significant correlations between all three conditions, the strongest correlation was observed between normalcy scores on DAF50 and normal speech, followed by the two DAF conditions.

Condition	DAF200	DAF50	No delay
DAF200	—	r = 0.52	r = 0.32
DAF50	r = 0.52	—	r = 0.91
No delay	r = 0.32	r = 0.91	—

### B. Speech production

**FIG. 2.**
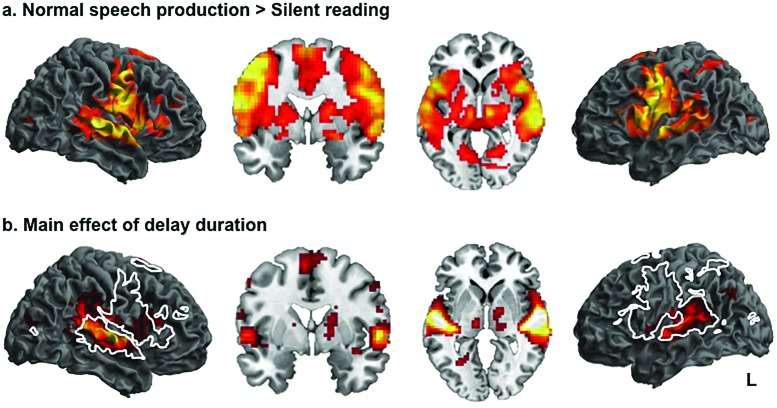
Speech production and main effect of delay is associated with activity in fronto-parieto-temporal networks in both hemispheres. Normal speech production was associated with widespread activity in dorsolateral temporal, somatosensory, primary, and premotor cortices in both hemispheres, as well as smaller clusters of activity in occipital lobe (a). Significant clusters showing a main effect of Delay are shown in (b). This revealed activity in a distributed network including superior temporal gyri, inferior parietal and frontal cortices in both hemispheres, with the strongest effect in the right. The majority of activity seen as an effect of delay lay within the general speech production network (white line). Activations are shown at a threshold of p < 0.005, with a voxel threshold of 20.

Compared to the baseline condition of silent reading, speech production was associated with widespread activity in dorsolateral temporal lobes, extending into parietal cortices and ventral and medial motor regions in both hemispheres with two smaller clusters in the occipital cortices [Fig. 2(a), p < 0.005, cluster threshold 20]. Within these large clusters, peaks lay in postcentral gyri corresponding to Brodmann areas 4 and 44, and in area TE3 of superior temporal gyrus [for coordinates see Table II(b)].

**TABLE II.** Significant peaks of BOLD activity in contrasts of interest. Peak coordinates of significant clusters are reported in Table II, with corresponding z and t scores, cluster size and anatomical labels. Coordinates are in mni space, all peaks were localized using the Eickhoff atlas (Eickhoff *et al*., 2005) which is available within SPM8.

Anatomy		Probability	k	f score	z score	x	y	z
(a) Main effect delay								
R superior temporal gyrus			2486	82.01	7.59	60	−16	4
R superior temporal gyrus	Area TE 1.1	43.5		59.17	6.93	45	−25	7
R superior temporal gyrus	Area PF (IPL)	55.8		53.68	6.73	66	−37	13
L superior temporal gyrus			1590	57.57	6.87	−51	−19	1
	Area Ig1	15.3		53.07	6.71	−30	−28	7
L superior temporal gyrus	Area PFcm (IPL)			45.85	6.41	−48	−37	19
L thalamus	Premotor	56.8	85	23.84	5.07	−15	−19	7
	Prefrontal	56						
L thalamus	Parietal	26		7.78	2.97	−12	−25	−5
L thalamus	Prefrontal	60.8		6.81	2.75	−15	-4	10
L posterior-medial frontal			202	17.24	4.43	−6	2	61
R posterior-medial frontal				10.57	3.51	6	11	70
R superior frontal gyrus				9.26	3.27	21	8	64
R thalamus	Prefrontal	58.8	201	16.38	4.33	9	−7	7
R thalamus	Prefrontal	29.6		14.92	4.15	18	−4	4
R caudate nucleus	Premotor	1.2		13.05	3.9	18	−4	16
			77	14.97	4.16	−27	14	16
L insula lobe				11.71	3.7	−27	20	7
L IFG (p. triangularis)	Area 44	15.9		9.7	3.36	−39	20	10
L angular gyrus	Area PGp (IPL)	48.8	115	14.5	4.1	−45	−70	34
L middle occipital gyrus	Area 7 A (SPL)	0.8	61	13.27	3.93	−27	−70	40
			42	13.05	3.9	−54	−7	52
L postcentral gyrus	Area 4 p	25.9		6.92	2.78	−45	−13	40
	Area hOc1 [V1]	13.3	37	12.74	3.85	−30	−61	1
R middle occipital gyrus	Area hOc4lp	32.6	155	12.71	3.85	42	−82	16
R middle occipital gyrus	Area hOc4lp	30.1		9.32	3.29	33	−88	22
R middle occipital gyrus				9.2	3.26	33	−70	34
R precentral gyrus	Area 4 p	8.5	53	11.95	3.73	42	−7	37
R precentral gyrus	Area 44	12.5		7.43	2.9	54	−1	43
L MCC			52	8.8	3.19	−3	−46	37
L MCC				8.29	3.08	−3	−34	37

Anatomy		Probability	k	t score	z score	x	y	z
(b) DAF0 > rest								
L postcentral gyrus	Area 4 p	28.8	12862	9.44	5.68	−57	−7	28
L postcentral gyrus	Area 44 s	36.9		9.31	5.65	−57	2	19
R superior temporal gyrus	Area TE 3	48.6		9.3	5.64	66	−28	1
L middle frontal gyrus			53	3.82	3.25	−33	41	22
L middle frontal gyrus				3.2	2.82	−39	35	28
			32	3.55	3.07	−21	−25	52
L precentral gyrus	Area 4 a	31.9		3.51	3.04	−21	−25	64
Anatomy		Probability	k	t score	z score	x	y	z
(c) DAF200 vs DAF50								
R supramarginal gyrus	Area PFm (IPL)	33.7	927	7.19	4.94	57	−40	25
R middle temporal gyrus				6.24	4.55	57	−28	1
R superior temporal gyrus				5.78	4.34	51	−37	10
L thalamus	Premotor	56.8	87	5.9	4.39	−15	−19	7
	Prefrontal	56						
L superior temporal gyrus	Area TE 3	19.2	230	5.44	4.17	−63	−40	13
L middle temporal gyrus	Area TE 3	2		4.93	3.91	−60	−34	7
L superior temporal gyrus	Area OP1 [SII]	13.9		3.97	3.34	−48	−34	10
	Area PFcm (IPL)	13.8						
L posterior-medial frontal			152	5.27	4.09	−6	2	61
R superior frontal gyrus				3.66	3.15	21	8	67
R posterior-medial frontal				3.65	3.14	12	2	64
R thalamus	Prefrontal	58.8	228	4.96	3.92	9	−7	7
R caudate nucleus				4.55	3.69	15	5	13
R thalamus	R caudate nucleus			4.03	3.38	15	−25	7
			52	4.86	3.87	−54	−7	52
L precentral gyrus				3.19	2.82	−42	−1	61
L insula lobe			94	4.84	3.86	−27	20	7
				4.48	3.66	−27	14	19
L insula lobe	Area 44	12.8		3.25	2.86	−39	17	7
L rolandic operculum	Area 44	33.7	42	3.84	3.26	−51	8	1
L IFG (p. opercularis)	Area 44	35.1		3.37	2.95	−60	5	13
R precentral gyrus	Area 4 p	8.5	24	3.61	3.11	42	−7	37
R calcarine gyrus	Area hOc1 [V1]	18.3	23	3.5	3.03	24	−73	10
R lingual gyrus	Area hOc1 [V1]	63.8		3.19	2.82	21	−64	1
R calcarine gyrus	Area hOc1 [V1]	75.2		3.18	2.81	18	−73	4
Anatomy		Probability	k	t score	z score	x	y	z
(d) DAF50 vs DAF200								
L angular gyrus	Area PGp (IPL)	70	187	4.94	3.91	−48	−73	37
				4.56	3.7	−39	−64	34
Anatomy		Probability	k	t score	z score	x	y	z
(e) DAF50 > DAF0								
R superior temporal gyrus	Area PFcm (IPL)	1.4	1554	10.13	5.87	45	−37	13
R superior temporal gyrus	Area PFcm (IPL)	22.4		9.94	5.82	54	−31	13
R superior temporal gyrus	Area TE 3	2.8		9.7	5.76	60	−16	1
L superior temporal gyrus	Area TE 1.0	14.1	1347	9.16	5.6	−51	−16	1
L heschls gyrus	Area Ig1	59.3		8.43	5.38	−33	−25	7
L superior temporal gyrus				8.3	5.33	−39	−40	22
L postcentral gyrus			48	4.12	3.44	−63	−7	37
L postcentral gyrus	Area 4 p	25.9		3.25	2.86	−45	−13	40
L postcentral gyrus	Area 1	11.1		3.12	2.77	−54	−10	46
Anatomy		Probability	k	t score	z score	x	y	z
(f) DAF200 ParamModn								
R putamen			146	5.05	3.75	30	−4	−2
R rolandic operculum	Area OP3 [VS]	15.8		4.34	3.4	54	−4	13
R insula lobe				3.56	2.95	42	2	−5
Cerebellar vermis	Lobule I IV (Hem)	46.4	36	4.94	3.7	0	−40	−23
L calcarine gyrus			68	4.69	3.58	−9	−55	7
L cerebellum	Lobule VI (Hem)	69.6	39	4.32	3.39	−21	−46	−23
L cerebellum	Lobule VI (Hem)	90		3.46	2.89	−18	−55	−17
	Area OP3 [VS]	54	33	4.1	3.27	33	−13	16
R postcentral gyrus	Area 3 b	49	32	3.52	2.93	51	−13	28
R precentral gyrus	Area 4 p	8		3.06	2.64	45	−7	37
Anatomy		Probability	k	t score	z score	x	y	z
(g) DAF50 ParamModn								
L lingual gyrus	Area hOc1 [V1]	50.9	39	5.24	3.84	−18	−79	1
L lingual gyrus	Area hOc4v [V4(v)]	26.8	44	4.44	3.45	−21	−61	−11
L lingual gyrus	Area hOc4v [V4(v)]	25.3		4.38	3.42	−24	−67	−5
R parahippocampal gyrus	Subiculum	54.7	71	4.33	3.39	21	−25	−14
R parahippocampal gyrus	Subiculum	34.9		4.18	3.31	27	−34	−11
				3.69	3.03	9	−19	-20
L IFG (p. triangularis)			30	3.94	3.18	−33	38	−2
L IFG (p. orbitalis)				3.84	3.12	−42	35	−5
R lingual gyrus	Area hOc1 [V1]	11.2	40	3.85	3.13	18	−73	−2
R calcarine gyrus	Area hOc1 [V1]	57.8		3.72	3.05	12	−85	−2
Anatomy		Probability	k	t score	z score	x	y	z
(h) DAF0 ParamModn								
R precentral gyrus			95	4.35	3.4	18	−19	58
R precentral gyrus	Area 4 p	26		4.32	3.39	24	−28	58
R paracentral lobe	Area 4 a	16		4.3	3.38	12	−25	58
R superior parietal lobule	Area 5 L (SPL)	64.2	49	4.3	3.38	18	−52	64
R precuneus	Area 3 b	28		3.98	3.2	12	−46	64
R superior parietal lobule	Area 7	23		3.7	3.04	30	−49	70

### C. Speech produced under different degrees of DAF

An ANOVA showed that the main effect of delay duration (200, 50, or 0) was associated with large clusters of activity in the temporal lobes in both hemispheres extending from mid to posterior STG and into ventral sensorimotor cortices and separate clusters in inferior frontal gyri and posterior parietal cortex [Fig. 2(b)]. These large clusters contains many peaks, including area TE of the superior temporal cortex, and PF and PGp of the inferior parietal lobe, left insular cortex, areas 4 p and 44 of the frontal cortices in both hemispheres, as well a multiple thalamic sites corresponding to prefrontal and premotor thalamus [see Table II(a) for details].

### D. Speech produced with different levels of delay

**FIG. 3.**
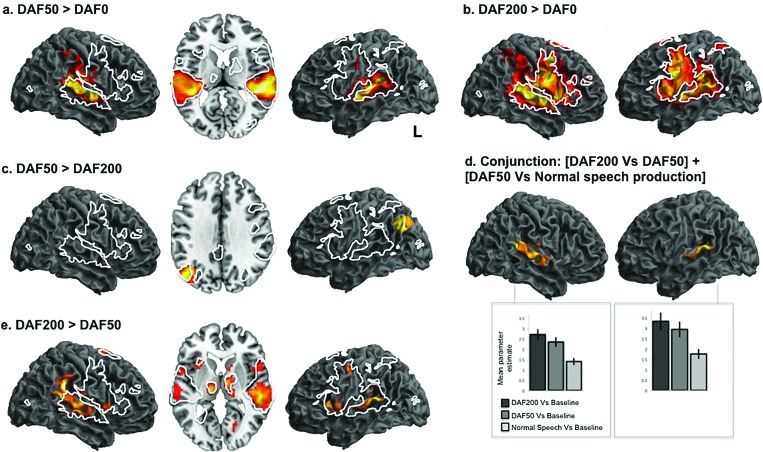
Neural networks engaged during speech production under DAF. (a)–(e) display patterns of BOLD responses revealed by comparison of different speaking conditions (DAF0, DAF50, and DAF200). The white lines depict the speech production network as identified in Fig. 2(a). In order to investigate where in the brain was sensitive to the amount of delay more than during normal speech production, we used a null conjunction of [DAF200 vs DAF50, (e)] and [DAF50 vs normal speech production, (c)] to look at voxels that significantly active in both contrasts [(d)—masking threshold p < 0.001]. This analysis revealed significant activity in bilateral superior temporal regions. Mean parameter estimates were extracted from the clusters revealed by the null conjunction and these are plotted in (d) (cluster peaks: −39 −37 7, −54 −22 7, −63 −40 13, 51 −37 10). Plots show parameter estimates (± 1 standard error of the mean). Activations are shown at a threshold of p < 0.005, with a voxel threshold of 20.

Comparing BOLD responses during both DAF conditions with speech under normal feedback condition revealed significant activity in widespread temporal, parietal and frontal regions in both hemispheres [white line, Figs. 3(a) and 3(b)]. Activity for DAF200 and DAF50 lay in similar regions of the dorsolateral temporal cortex, but for DAF200, activity spread into inferior frontal regions.

A direct comparison of the two DAF conditions (DAF200 > DAF50) revealed significant activity in bilateral superior temporal gyri (TE3), more so on the right, extending posteriorly into the supramarginal gyrus of the inferior parietal lobe, corresponding to area PFm, and SII (OP1) [Fig. 3(e)]. With the exception of the right IPL activity, these peaks almost entirely lay within regions that are sensitive to speaking under normal feedback condition (white line, [DAF0 > silent reading]).

Other peaks identified by this contrast lay in the inferior frontal gyri (BA 44) in both hemispheres, left insula, left pre- and post-central gyri, pre-supplementary area, calcarine sulcus (BA 17/ hOc1[V1]) and subcortically in the basal ganglia corresponding to premotor/prefrontal thalamic sites. For full details of peaks and subpeaks, see Table II(c). The reverse contrast revealed a single cluster of activity in left angular gyrus of the posterior parietal cortex corresponding to PGp [Fig. 3(c), Table II(d)].

The comparison of speaking under a minimal but noticeable delay compared to normal feedback (DAF50 > DAF0) was associated with widespread activity in bilateral superior temporal gyri corresponding to TE1, TE3, and left Heschl's gyrus (Ig1). These clusters extended into inferior parietal cortices in both hemispheres, with peaks lying in PFcm, and into post central gyrus which maps to somatosensory cortex [Fig. 3(a), Table II(e)].

In order to look at where regions that are sensitive to the amount of delay, over and above the response to speech production under normal feedback, a null conjunction of [DAF200 > DAF50] and [DAF50 > DAF0] was carried out. This analysis revealed voxels that are significantly active in both contrasts in superior temporal regions in both hemispheres with a more distributed pattern on the right (p < 0.005, cluster threshold 20). These STG clusters were used to create regions of interest from which mean parameter estimates were extracted. These are plotted in Fig. 3(d) (the peaks within these clusters were −39 −37 7, −54 −22 7, −63 −40 13, 51 −37 10).

### E. Behavioral scores of speech output

A one way repeated measures ANOVA revealed significant differences between the scores assigned to sentences spoken under the three different conditions of DAF200, DAF50, and no delay [F(2,42) = 42.852, p < 0.005] means/standard deviations 3.59 +/− 1.941, 5.14 +/− 2.022, and 8.20 +/− 1.182, respectively. A Games-Howell *post hoc* test revealed that the scores for all three conditions are significant different from each other.

A product-moment correlation coefficient was computed to assess the relationship between mean scores across all three conditions. This approach revealed significant correlations between the subject mean scores on the three conditions. Speech produced under normal feedback and under DAF50 conditions was highly correlated [r = 0.91, n = 15, p < 0.05) indicating a strong positive relationship between ability to produce normal speech under DAF50 and normal sounding speech under normal feedback conditions. Subject mean ratings of speech produced under DAF200 were correlated with DAF50 speech ratings [r = 0.52, n = 15, p < 0.05) indicating a moderate positive relationship between ability to produce normal speech under the two delay conditions. Speech produced under normal feedback and under DAF200 conditions was the least correlated [r = 0.32, n = 15, p < 0.05) indicating only a weak to moderate relationship between normal sounding speech under no delay and the delay with maximal interference. This indicates that subjects who produced fluent, clear speech under conditions of no delay, also tended to produce more normal sentences under both the DAF conditions (see Table I).

### F. Within-subject ability to produce normal sounding speech across all conditions

**FIG. 4.**
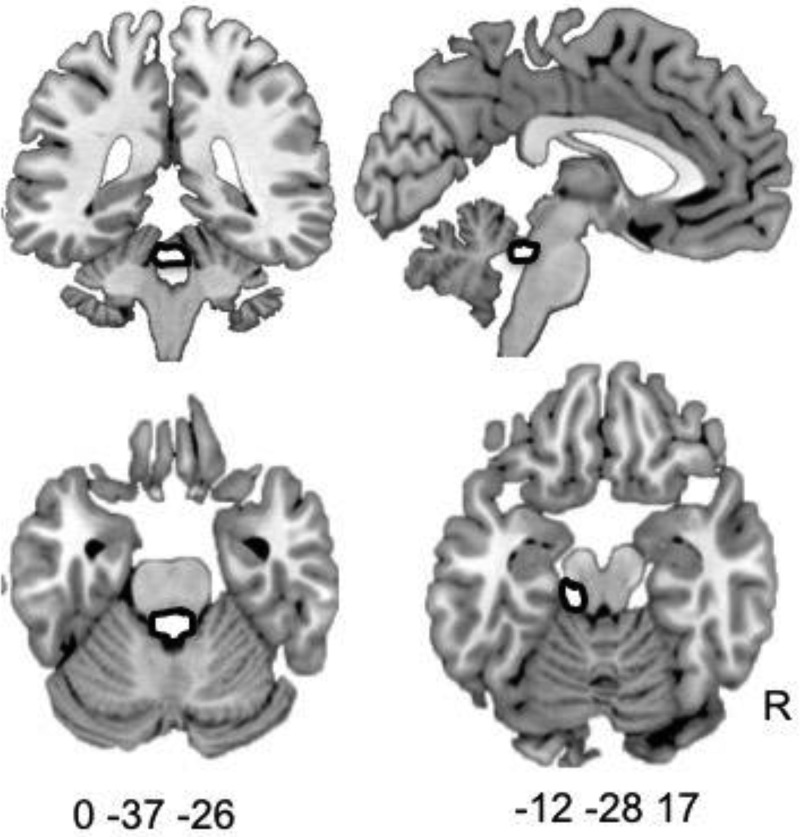
Production of normal sounding speech is associated with activity in periaqueductal grey. In all speech production trials (DAF 200, DAF50, and normal feedback), verbal output was recorded and rated for normalcy. Mean normalcy scores, as rated by three raters, were entered into a first level model on a trial-by-trial basis in order that the neural correlates of normal sounding speech production within subjects could be identified at the second level. This approach revealed significant activity in midline brain stem structures corresponding to the periaqueductal grey. Two separate PAG peaks were observed, one on the midline (top two and bottom left panel) and one lying more dorsally and to the left (bottom right panel, p < 0.005, cluster threshold 20). Coordinates are reported in mni space.

The first approach to looking at individual differences in speech production was to look at where BOLD responses correlated with each subject's individual performance across conditions in terms of normalcy ratings, across all speech production conditions. In order to do this a single regressor was made comprising each subject's mean normalcy scores (average normalcy score, as rated by three raters) on a trial-by-trial basis. These were entered into a second level model revealing two significant peaks of activity in the midline and left midbrain corresponding to the periaqueductal grey (PAG) (Fig. 4, p < 0.005, cluster threshold 20). These activations were localized using two human MRI atlases (Afshar *et al.*, 1978; Naidich *et al.*, 2009).

### G. Across subject ability to produce normal speech under various DAF conditions

The second approach used to look at variability was to look at where in the brain BOLD responses correlated with mean performance in producing fluent speech, under different feedback conditions (200, 50, and 0 ms). This allowed us to explore the relationship between high normalcy performance and BOLD responses during speech produced under these different DAF conditions.

In order to do this, for each DAF condition, a mean score was calculated for each subject and was entered into a second level model for the contrast of speaking under DAF (200, 50, and 0 ms separately) compared to passive listening. Passive listening was used as the baseline in this analysis in order to control for any differences in the auditory processing.

**FIG. 5.**
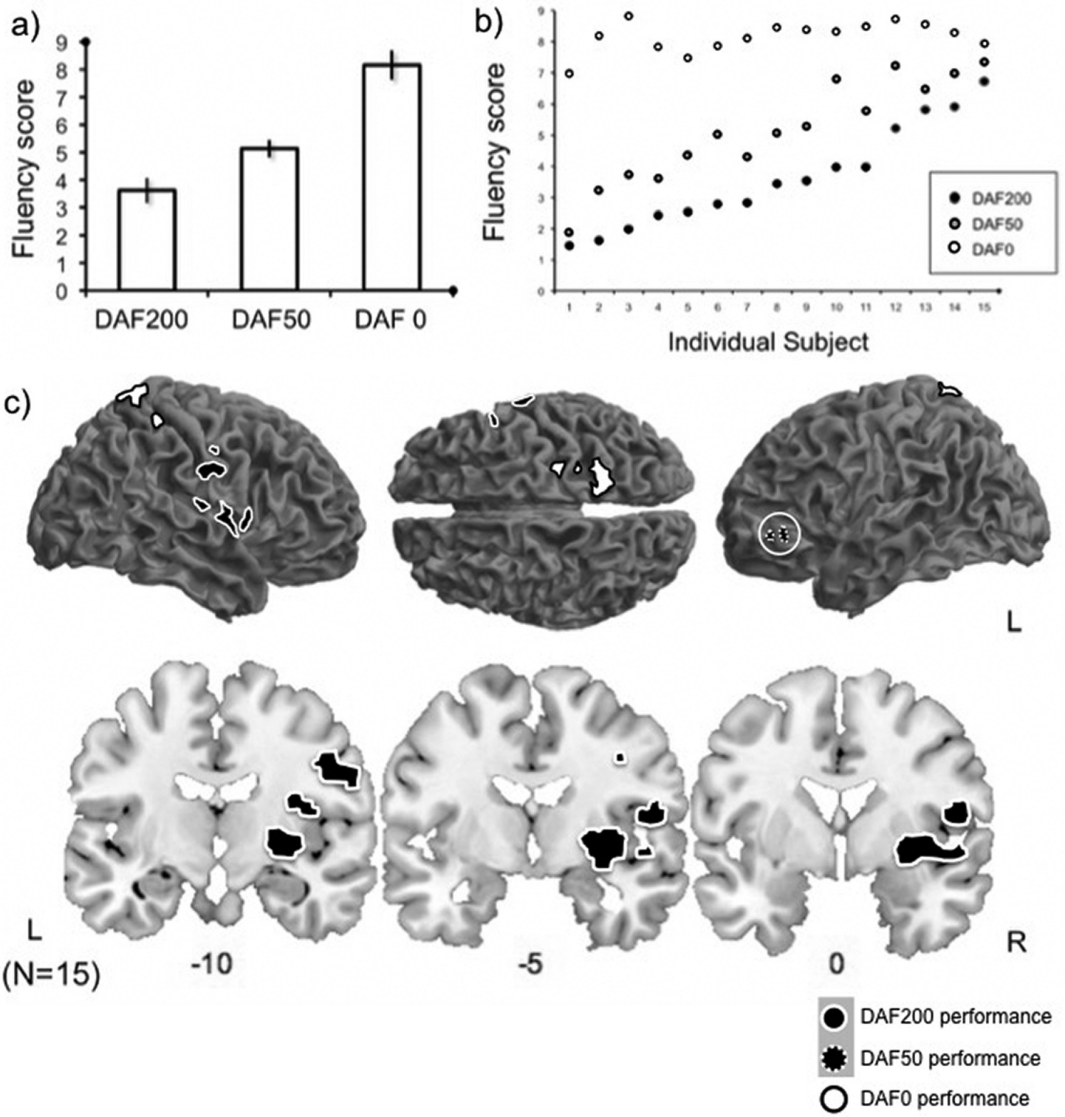
Higher scores in speech production under 200 ms DAF are associated with significant activity in ventral somatosensory, motor, insula cortex and bilateral parietal operculum. Mean fluency scores (a) for the three speech conditions were significantly different from each other [F(2,42) = 42.852, p < 0.001], means/standard deviations (DAF200 = 3.59 +/− 1.94, DAF50 = 5.14 +/− 2.02, DAF0 = 8.20 +/− 1.18). There was a high level of variability in performance between subjects but performance in the three conditions was correlated, most strongly so between speech produced under DAF50 and normal feedback delayed auditory feedback conditions. In order to look at where performance is correlated with neural activity (within subjects), means scores for each trial were used as a parametric modulator. Under DAF200 conditions, this approach revealed significant activity in right ventral somatosensory cortices spreading into motor cortex, putamen insula cortex, parietal operculum [(c), black filled, white outline]. The two peaks in the insula cortex lie in dorsal and ventral posterior insula, subcortical peaks are listed in Table II. The same approach for speech produced under a 50 ms delay revealed significant activity in left inferior frontal gyrus (black filled, dotted white outline) and for normal speech production we saw activity in right superior parietal cortex (white filled, black outline, p < 0.005, cluster threshold 20).

Higher ratings of speaker normalcy under DAF200 conditions were positively correlated with significant activity with two peaks in right insula cortex, right putamen, and ventral somatosensory and motor areas which map to OP3, BA3b, and BA4p, and lobule VI of the left cerebellum [Fig. 5(c), subcortical peaks are listed in Table II(f)]. In contrast, higher ratings of speaker normalcy under DAF50 conditions were correlated with activity in left inferior frontal gyrus (IFG) [Fig. 5(c)]. Thus, here we show that independent of performance, the networks generally active during DAF50 lie within areas activity during DAF 200 (Fig. 3). However, ability to produce fluent sounding speech under these two feedback delays is associated with activity in distinct regions (Fig. 5). Finally, trials on which speech was rated as highly normal under no delay were associated with activity in right superior parietal cortex [Fig. 5(c)]. The reverse contrast reflecting a negative relationship between ratings of speech production and BOLD activity revealed no significant activations (p < 0.005, cluster threshold 20).

## IV. DISCUSSION

This study investigated variability in the ability to produce normal sounding vocalizations under minimal and maximal feedback delays. Previous work has repeatedly shown that a 200 ms delay in auditory feedback during speech production results in maximal dysfluency, but that individual variability in response is high. Here, for the first time, we look to see how BOLD responses correlated with ability to produce normal sounding speech, under 50 and 200 ms feedback delays.

Independent of variability in susceptibility to DAF, we confirm previous findings that altered auditory feedback during speech production is associated with activity in posterior superior temporal cortices (Hashimoto and Sakai, 2003; Takaso *et al.*, 2010), and that speech production under 200 and 50 ms delays lie largely within in a region activated with speech production in general. This is consistent with previous work showing that peaks of STG activity observed during DAF are sensitive to length of delay. In this study, only one region in left in left posterior temporal parietal junction was active for increasing delay, but not delayed feedback compared to no delay (Takaso *et al.*, 2010). Speaking under DAF conditions was associated with significant activity in bilateral superior temporal gyri. Supported by findings in non-human primates (Eliades and Wang, 2008), a prominent model of speech production (Guenther *et al.*, 2006) suggests STG neurons encode error between the predicted and actual auditory consequences of a vocalization. We report that even when performances is co-varied out, activity is still seen in STG. Given that on trials when subjects produce more “normal” speech under DAF, they are producing auditory vocalizations closer to their target sound/auditory template, STG activity might be predicted to diminish with superior performance. Thus these data tentatively suggest that either STG may be encoding something other than, or as well as, error, e.g., in detecting and compensating for the amount of delay (Takaso *et al.*, 2010).

### A. Susceptibility to different delay durations is associated with activity in different regions

We report that scores for individuals who produce normal sounding speech under 200 and 50 ms delays are correlated, yet are associated with partially distinct patterns of peak activity: The former comprising right putamen, and ventral motor, somatosensory, insula and parietal opercular cortices, and the latter with activity in left IFG. The role of these two distinct networks may reflect different strategies adopted in order to overcome the different DAF conditions, rather than a unitary dimension of difficulty, recruiting one neural system to a greater or lesser degree. Furthermore, we report for the first time that the production of normal sounding speech across all conditions is positively associated with activity in the periaqueductal grey, a region commonly implicated in production of vocalizations (Larson, 1988). These data indicate that both motor and somatosensory regions, as well as subcortical structures, may be recruited to support speech production under altered auditory feedback.

The production of normal sounding speech under 200 ms revealed significantly greater activity in a range of areas within the speech production network. This is in accordance with previous research on DAF (Watkins *et al.*, 2005), and with recent data showing that intersubject variability in neural activity is evolutionarily meaningful and tends to be higher in association cortices and cortical regions where individual differences in cognition occur (Williams *et al.*, 2001). The left IFG and neighboring insula cortex, is significantly active during normal speech production whereas the right IFG is deactivated during propositional speech (Blank *et al.*, 2003). Here, we show that under difficult conditions this profile is reversed. Insula cortex has been implicated in a number of cognitive processes relevant to speaking under DAF. First, anterior insula plays a crucial part in speech production (Dronkers, 1996; Borovsky *et al.*, 2007), in the control of articulators rather than pre-articulatory planning (Ackermann and Riecker, 2004), however, speech and language areas of the insula are thought to lie rostrally to those reported here (Kurth *et al.*, 2010). According to this meta-analysis, the peaks reported here were delineated as sensorimotor insula cortex activated by interoception. For example, activity in the insula cortex has been implicated in encoding limb ownership (Tsakiris *et al.*, 2008) and ownership of action (Farrer *et al.*, 2003) and insula damage is associated with anosognosia (Karnath *et al.*, 2005). Here, we show that during trials in which people perform well under DAF conditions there is increased activity in right insula cortex, and it is possible that there is some interaction between ownership of the reafferent feedback (source encoding) and performance. This requires further investigation.

The basal ganglia have a well known role in motor function, both in normal speech and more recently in reward driven motor learning (Doya, 2000). A recent study has demonstrated a clear role for the basal ganglia in vocal learning in the songbird (Warren *et al.*, 2011). They report that the kind of learning that occurs in the pathway from the basal ganglia to the premotor cortex is a gradual process. The current data demonstrate that an ability to perform well under DAF conditions also engages aspects of the basal ganglia indicating that there may be an element of motor learning underlying subjects' performance. In normal speech, basal ganglia activity is thought to reflect production unit selection and sequencing (Ghosh *et al.*, 2008). Both of these processes are likely involved in the production of normal speech under increasingly difficult feedback conditions. It is possible then that the increased activity in basal ganglia observed here may reflect the increased selection and sequencing processes that underlie the production of more fluent speech.

We also found peaks in primary motor and somatosensory cortices and cerebellum associated with normal sounding speech under a 200 ms feedback delay. Recent work (Pruszynski *et al.*, 2011) has demonstrated a causal role for neurons in primary motor cortex in the integration of information about movement of multiple effectors (elbow and shoulder). Thus, it has been suggested that primary motor cortex is a candidate for the integration of voluntary and feedback control (Franklin and Wolpert, 2011). It is possible that in higher performance trials, subjects are better at integrating reafferent information from multiple parts of the articulators in different or more efficient manner. Previous studies of adaptation and variability in dealing with altered sensory consequences of action have reported a role for the cerebellum as an adaptive filter. We observed two peaks of cerebellar activity, one in the midline vermis and one in the left cerebellar hemisphere, corresponding to lobule IV/V. We did not acquire data across the entire cerebellum and thus cannot comment further on how the present data relate to cerebellar function.

Producing normal speech under a 50 ms delay was associated with activity in the left IFG, a region that has been linked to individual differences selective in response inhibition (Forstmann *et al.*, 2008; Swick *et al.*, 2008). It is likely that producing speech under a 50 ms delay provides enough interference to engage these response inhibition systems in a way that producing speech under 200 ms does not.

### B. The role of unreliable feedback in maintaining fluent speech under a delay

There are two main interpretations that persist as to why some subjects compensate for feedback altered speech under certain situations. On one hand, it has been suggested that DAF induced speech disruption indicates that speech relies on auditory feedback. When feedback is unreliable or noisy, it is hypothesized that motor control processes engage feedforward processes to compensate: for example, it has been suggested that individuals with poor control of pitch, shift their voice control from feedforward to feedback processes in order to detect errors and update their internal model accordingly (Scheerer and Jones, 2012). It is thought that when the internal model (the mapping between motor commands and reafferent information) is consistently accurate, feedforward processing dominates, and feedback processes are engaged only for the purpose of error detection (Civier *et al.*, 2010). This context appropriate weighting of the ratio of feedback and feedforward processes may be central to successful motor control. Here, we show that minimally and maximally interfering temporal delays (between speaking and hearing), engage different aspects of the sensorimotor speech control system. Further work should aim to specifically explore the role of these regions in feedforward and feedback vocal motor control.

### C. The role of attention in maintaining fluent speech under a delay

An alternative explanation of the DAF effect on speech, is that DAF forces speakers to attend to their own reafferent feedback to a disruptive degree, and that they then modulate their speech to counteract any distortion (Borden, 1979). In support of this interpretation, it has been shown that the speed of speech influences the number of errors made under DAF: Zanini and colleagues (1999) show that whilst speakers producing speech under a 200 ms delay always produce more errors than under no delay, increasing the speed of their speech under 200 ms delays reduced their error rate. They suggest that increasing speech rate engages central mechanisms of movement programming and attentional control via cortico-cerebellar loops more than sensory feedback systems, resulting in less DAF induced speech errors. These authors also found that speech errors were greater when the auditory input was returned to the right ear independent of delay duration or speaking rate, which they interpret as evidence that the left hemisphere is more susceptible to DAF, suggesting a possible role for hemispheric specialization in susceptibility to DAF. Here, we show that higher ratings of speaker normalcy under a 200 ms delay were positively correlated with significant activity in two peaks in right cortices, which is in accordance with their suggestion that the left hemisphere is more susceptible to DAF. By comparing EMG activity during DAF, Borden *et al.* (1976) were able to show an irregular relationship between specific muscle EMG under normal and delayed feedback conditions, even though the delay in auditory feedback remained constant. They interpret this as evidence against an error monitoring interpretation, in which they expect to see a consistent relationship between motor output and feedback delay. Instead they consider their data to suggest attentional mechanisms at play which change over time. Thus, it is possible that the variability we report here is due at least in part to attentional mechanisms, and that the differences we see in ability to produce normal sounding speech under different delay conditions, relates to differences in the attentional resources employed by these different delay conditions. However, the lack of increased activity in prefrontal (Cohen *et al.*, 2000) or parietal (Rushworth *et al.*, 2001) regions suggests superior performance seen here cannot be accounted for just by increased attentional processing.

There is a high degree of individual variability in adaptation to altered auditory feedback (Houde and Jordan, 2002) and evidence suggests that adaptation occurs in the first few hundred milliseconds of exposure (see Shadmehr *et al.*, 2010). Tiffany and Hanley (1956) found that highly susceptible subjects had a slower rate of speech subsequent to DAF exposure, and those least affected, a faster rate. It has been suggested that individuals who perform well are able to use somatosensory feedback, where as high susceptibility individuals are dependent on auditory feedback (Yates, 1963; Attanasio, 1987), however, others have failed to find supporting evidence (Burke, 1975). We report a correlation between performance under DAF 200 and normal feedback, however, there is little evidence for a correlation between ability to produce normal speech under DAF and other psychological or language abilities (Arens and Popplestone, 1959). Speaking faster is known to reduce performance (Stuart *et al.*, 2002), and while slowing of speech reduces stuttering it also may change the peak interference delay (MacKay, 1968) indicating that it is not just the length of a speech sound that causes the problem.

### D. Across all delay durations, producing fluent speech is associated with activity in periaqueductal grey

The role of the periaqueductal grey in the production of vocalizations has been well described in non-human animal models (Jurgens, 1994): periaqueductal grey neurons begin firing before the onset of vocalization, indicating a role beyond feedback processing (Larson, 1988), while lesions to this region results in mutism (Esposito *et al.*, 1999) without akinesia (Jurgens, 1994). Periaqueductal grey neurons, indirectly connected to phonatory motor neuron pools are thought to serve a dual role, coordinating phonatory muscles and linking sensory information and motivational levels (Jurgens, 1994). Here, we show for the first time that production of normal sounding speech is associated with increased activity in the periaqueductal grey, across all three manipulations to auditory feedback. We consider this a tentative finding, which warrants further work to confirm this effect, as imaging the PAG is notoriously difficult due to motion artifacts and noise from cardiac movement.

### E. Considerations and limitations

Finally, despite the wide variation in the types of changes to speech that occur under DAF, the current study collapsed across all types of speech errors. With 20 subjects there is not sufficient statistical power to tease apart the different strategies adopted. This is of great interest, however, and we hope that future studies will be directed at trying to dissociate between the different approaches to dealing with interference from DAF during production of vocalizations. It is worth pointing out that the current study is unable to distinguish between the neural structures responsible for coping with producing normal sounding speech under DAF, or those that encode some downstream consequence of applying certain strategies.

### F. Conclusion

In conclusion, we report a high level of inter-subject variability in susceptibility to the effects of DAF. Within subjects, production of normal sounding speech across all conditions was associated with subcortical structures known to play a key role in vocalization. Subjects produced speech that was rated as significantly worse under a 200 ms delay compared to a 50 ms delay. In accordance with this behavioral dissociation, the ability to produce normal speech under these two delay conditions was associated with increased activity in different neural networks, suggesting a differential neural sensitivity to the magnitude of temporal shifts in feedback. We show that independent of performance, the networks generally active during DAF50 lie within areas activity during DAF200. However, the ability to produce fluent sounding speech under these two feedback delays, are associated with activity in distinct regions. This might reflect the use of distinct strategies in dealing with speech production under a 200 or 50 ms delay. These data demonstrate the key roles of both cortical and subcortical structures in producing normal sounding vocalizations, and that distributed aspects of a sensorimotor network comprising both cortical and subcortical structures are engaged when overcoming the interfering effect of a 200 ms delay. Future work may aim to elucidate whether these data reflect correlates of a pre-existing characteristics, an adaptive coping strategy, or a form of motor learning employed by certain individuals.
